# Pseudoaneurysm Leading to Aortic Dissection: An Interesting Case Presentation

**DOI:** 10.7759/cureus.8545

**Published:** 2020-06-10

**Authors:** Liang D Ge, Woosun Kang, Puja Patel, Raj Patel, Tinoy Kizhakekuttu

**Affiliations:** 1 Internal Medicine, University of Illinois College of Medicine at Peoria, Peoria, USA; 2 Internal Medicine, American University of Antigua, Peoria, USA; 3 Cardiology, University of Illinois College of Medicine at Peoria, Peoria, USA

**Keywords:** cardiology, cardiac imaging, vascular disease

## Abstract

This is an interesting case of ascending penetrating aortic ulcer (PAU) leading to pseudoaneurysm and eventually type A aortic dissection and peri-aortic hematoma. PAUs are common clinical manifestations, however, uncommonly lead to pseudoaneurysms that cause aortic dissection.

## Introduction

Vascular disease affects millions of patients in the United States annually. It is a leading cause of morbidity and mortality in addition to increased burden on healthcare costs. Progression of vascular disease generally starts with initial injury to the vascular endothelium, with subsequent formation of atheromatous plaque, followed by rupture, and finally to dissection or other acute pathological conditions. Within the various manifestations of vascular disease, penetrating aortic ulcers (PAUs) are common findings in patients with longstanding risk factors for atherosclerotic vascular disease [1]. However, PAUs can progress in the form of aneurysms, pseudoaneurysms, and even aortic dissection [2]. It is imperative for clinicians to recognize that PAUs can manifest into more aggressive and clinically significant aortic diseases including dissection. We present the case of an elderly female with a known history of penetrating ulcer which caused psuedoanuerysm formation and eventual dissection with peri-aortic hematoma.

## Case presentation

**Figure 1:**
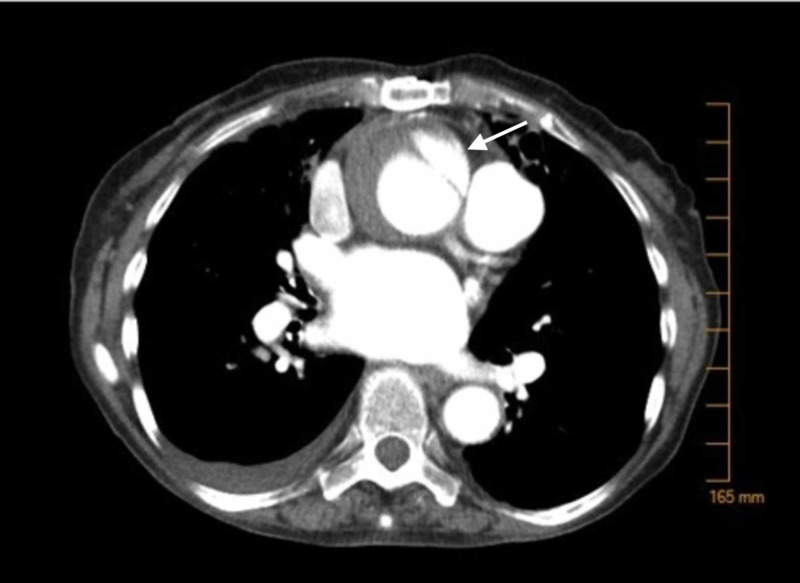
Arrow showing ascending aortic pseudoaneursym.

A 91-year-old female with a past history of mycobacterium avium complex infection, heart failure with preserved ejection fraction, prior transient ischemic attack, bronchiectasis, hypertension (HTN), hyperlipidemia (HLD), and obstructive sleep apnea initially presented to her outpatient cardiologist’s office with a two-week history of dyspnea on exertion, fatigue, and low-grade fevers. She has a history of atrial fibrillation status post Maze procedure in 2002, complicated by right-sided pleural effusion postoperatively. Her symptoms were initially suspected to be due to infection. The patient completed a course of doxycycline and ampicillin-clavulanic acid without significant improvement. A chest CT obtained for further evaluation showed an ascending aortic pseudoaneurysm with a 12 mm x 13 mm neck and a sac measuring 47 mm x 12 mm x 37 mm, likely secondary to penetrating plaque ulceration (see Figure 1).

Upon receiving CT results, the patient was admitted; she continued to endorse generalized fatigue, weakness, and shortness of breath. The patient was afebrile and denied any chest, back, or abdominal pain, nausea, vomiting, diarrhea, wheezing, or coughing. Vital signs were as follows: Temp. 98.1°F, heart rate (HR) 71, blood pressure (BP) 141/64, respiration rate (RR) 22, SpO2 94% on ambient air. On evaluation, the patient was deemed a poor candidate for surgical repair and replacement of the ascending aorta due to her advanced age, multiple co-morbidities, and high likelihood of requiring a repeat procedure thus, the family and care team elected for medical management.

Blood pressure was optimized with losartan, enalapril, amlodipine, metoprolol, and hydralazine with the intended goal to maintain systolic BP < 130. She was concomitantly treated empirically for community acquired pneumonia with azithromycin and ceftriaxone, and discharged.

On follow up CT chest scan for bronchiectasis evaluation one year later, the patient was noted to have a dissecting aneurysm that had increased to 5.7 cm though remained asymptomatic and continued medical management was pursued with up-titration of hydralazine. 

**Figure 2:**
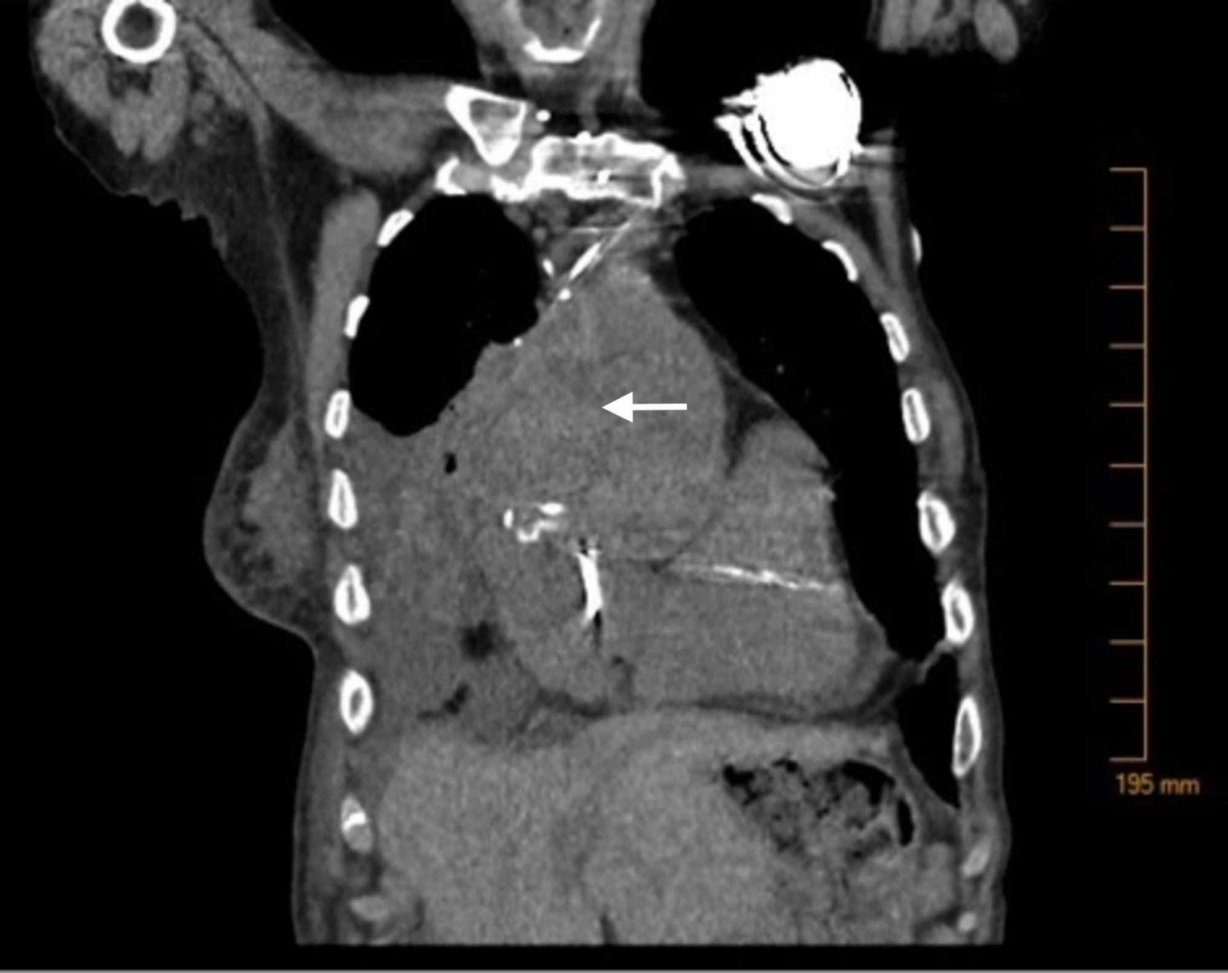
Arrow showing large peri-aortic hematoma formation in the setting of acute aortic dissection.

The patient subsequently presented to the ED one year later with complaints of mid-sternal chest pain. Vitals on presentation were as follows: Temp. 98.8°F, HR 89, BP 117/74, RR 20, SpO2 93% on room air. Labs were significant for a peak troponin of 24.9, brain natriuretic peptide (BNP) 228, and D-dimer 2.61. The electrocardiogram (EKG) was concerning for acute inferior wall myocardial infarction. An aortic root hematoma was identified on trans-thoracic echocardiogram and confirmed by chest CT (see Figure 2), likely caused by acute aortic dissection. The CT chest scan showed the aneurysmal sac size had increased to 9 cm. After discussion of options with the patient and family, she was eventually discharged to hospice and passed one month later.

## Discussion

This case represents an uncommon sequelae of aortic atherosclerotic disease and presents a good illustration of natural disease progression. A penetrating aortic atherosclerotic ulcer is defined by an atherosclerotic plaque that extends through the internal elastic lamina of the media layer of the aortic wall, thereby allowing hematoma formation within the intima media [1]. This case contrasts a classic aortic dissection, which is caused by an intimal tear at a point of maximal hydraulic stress [3]. With the advancement of computed tomography angiography (CTA), it is possible to diagnose PAU with imaging alone, without the need for histopathological diagnosis, as was seen in this case [2]. It is estimated that approximately 2%-7% of patients with symptomatic aortic disease will have penetrating atherosclerotic ulceration [2, 4]. The most significant risk factors for PAU include old age, hypertension, and hyperlipidemia [2-4], all of which were present in our patient. Of patients with PAU, one study cited approximately 90% occurring in the descending aorta [2].

In another study, anterior chest or mid-scapular back pain was found to be the most common presenting symptom, although patients can be asymptomatic on initial presentation, like in our case. Complications of PAU include aneurysms, dissections, pseudoaneurysms, and aortic rupture [4]. In our patient, a pseudoaneurysm was noted as the likely manifestation of PAU which eventually led to dissection. In symptomatic patients, the rate of rupture is reported to be as high as 38%, although it is significantly lower in asymptomatic patients [2]. But while our patient was asymptomatic initially, her disease did eventually progress to acute aortic dissection with aortic rupture and hematoma. When PAU causes intramural hematoma formation of the ascending aorta, emergent surgical intervention is recommended due to risk of complications such as cardiac tamponade, aortic rupture, or compression of the coronary arteries [4].

This patient may have benefited from surgery if her aortic disease were isolated, however, several underlying co-morbidities made the patient a poor surgical candidate. For such patients, optimal medical therapy includes normotension and preload reducing medications [4]. Beta blockers and calcium channel blockers have been shown to have a significant mortality benefit, both of which were used in this patient [4].

## Conclusions

A common manifestation of penetrating ulcers is pseudoaneurysm formation which can uncommonly lead to aortic dissection. Patients who have established diagnoses of aortic penetrating ulcers and any sequelae of the condition (i.e. pseudoaneurysm as in our case) should be scrutinized for aortic dissection based on clinical presentation. Our hope is this case illustrates the importance of surveillance and aggressive risk factor modification/medical management of aortic penetrating ulcers.
